# Supplementary material for: A caveat to using wearable sensor data for COVID-19 detection: The role of behavioral change after receipt of test results
Source: PLoS One. 2022 Dec 30;17(12):e0277350. doi: 10.1371/journal.pone.0277350 (PMC9803125; doi:10.1371/journal.pone.0277350)
Supplement: Attachment — Submitted filename: ResponseToReviewers_PLOSONE_RR1.docx [file pone.0277350.s002.docx]

**Reviewer 1:**

1. **The rationale for this study alone is not particularly impactful. I am not sure why utilising a wearable for this purpose would be useful, given the proliferation of self-testing kits. Given that the positive sample in this study are symptomatic, it is expected that their behavior will change.**

We thank the reviewer for bringing up the proliferation of self-testing kits and rapidly available test results, and agree that testing is likely the most reliable way to detect infection. We also now highlight that all individuals in our sample were symptomatic, while cases test positive for COVID and controls test negative (might have the flu or a cold instead). This allows us to compare across two groups of individuals both feeling subjectively unwell and therefore both reasonably likely to engage in self-care behaviors, but the COVID-positive individuals were more likely to be quarantined and take extra self-care.

*Location: Page 5, Lines 118-147; 152-56*

2. **I understand that the main utility for COVID-19 detection using wearable devices is to make the wearer aware of changes to their physiology, so that they can seek testing and reduce the spread. The analysis of this data do not allow for this, and would not provide insight into how to reduce the spread of the virus. It can be covered by general health recommendations of "get tested when you have symptoms".**

This helpful comment from the reviewer highlighted the rationale for our study was not clear. We now have edited and reframed the introduction to emphasize that our primary goal is to urge caution in interpreting findings from the growing number of studies suggesting an important role for wearables in SARS-CoV-2 infection detection and alert researchers to a potential effect of test result receipt . We show that since no other studies provide information on test result receipt date, the current literature is unable to demonstrate conclusively that physiology change precedes test result receipt (which would be required in order to use wearables to alert users to seek testing). Rather, our results suggest that the signatures of COVID-positive wearable metrics are most pronounced AFTER the individual gets their test results, suggesting that an individual’s wearable metric change is dependent on the test result.

**Reviewer 2:**

1. **Include a list of four to ten key words after the Abstract**

Thank you for this guidance. We have included a list of keywords (wearable device, COVID-19 infection, COVID-19 detection, behavior change, model accuracy, public health, epidemiology) after the abstract.

*Location: Abstract, Lines 102-103*

2. **The aim is not clear in the abstract section; mention major contribution and paper organization clearly**

We are grateful to the reviewer for the feedback on clarifying the aims throughout the abstract and paper. We have updated the abstract, introduction, and discussion sections with a statement of our aims and main contributions. Specifically, our aim as well as the major contribution was being the first study to assess the role of COVID-19 test result receipt on wearable metrics’ discriminative capacity in distinguishing SARS-CoV-2 positive and negative symptomatic individuals.

*Location: Page 5, Lines 148-156*

3. **More recent literature survey required, and compare with them**

We appreciate the reviewer’s direction and have updated the introduction with a more recent literature review.

*Location: Page 4-5, Lines 106-156*

4. **Improve the results and discussion section, add more sentences for proper justifying the works**

We have updated the discussion section with contributions and justification for the study.

*Location: Page 16, Lines 336-347*

5. **Try to improve the usage of English grammar. The formatting, grammar and typo errors should be carefully checked before processing this article.**

We appreciate the suggestion and have checked and corrected the use of English grammar, formatting and typos throughout the manuscript.

6. **Measure accuracy of the model**

We agree the accuracy of the models are important and have used AUC and its 95% confidence interval as our primary measure of accuracy, noted in the Results text and in the Figures.

7. **Consider this paper Clinical and Laboratory approach to Diagnose COVID-19 using Machine Learning, Interdisciplinary Sciences: Computational Life Sciences, Intelligent Computing on Time-Series Data Analysis and Prediction of COVID-19 Pandemics, Pattern Recognition Letters**

We appreciate the reviewer’s suggestions of additional papers to review and appreciated their background on machine learning methods; however, as our paper focuses primarily on behavioral change with test result date inclusion and less on developing a prediction algorithm or model, we restrained our literature review to those papers directly related to wearables metrics and change with COVID-19 infection.

8. **Measure computational complexity**

We have included a sentence in Methods noting that these models run relatively quickly, on the order of milliseconds.

*Location: Page 12, Lines 239-240*

9. **Highlights major deliverables and future works**

We have updated the aims and discussion to emphasize major findings and potential future directions.

*Location: Page 5, Lines 148-152; Page 16, Lines 336-347; Page 17-18, Lines 383-396*

10. **Why you consider wearable approach for this work**

Given the large interest and increasing body of work in using wearables for passive, real-time physiological data collection in infectious disease monitoring, we felt that investigating what factors affect changes in wearables data during an illness is a prudent and impactful contribution to the wearables literature in this pandemic and future pandemics.

**Editorial/Production:**

**Journal Requirements:**

**1. When submitting your revision, we need you to address these additional requirements.**

**Please ensure that your manuscript meets PLOS ONE's style requirements, including those for file naming. The PLOS ONE style templates can be found at**

[**https://journals.plos.org/plosone/s/file?id=wjVg/PLOSOne_formatting_sample_main_body.pdf**](https://journals.plos.org/plosone/s/file?id=wjVg/PLOSOne_formatting_sample_main_body.pdf) **and**

[**https://journals.plos.org/plosone/s/file?id=ba62/PLOSOne_formatting_sample_title_authors_affiliations.pdf**](https://journals.plos.org/plosone/s/file?id=ba62/PLOSOne_formatting_sample_title_authors_affiliations.pdf)

We have formatted the manuscript following the requirements in the style templates.

**2. Please provide additional details regarding participant consent. In the ethics statement in the Methods and online submission information, please ensure that you have specified what type you obtained (for instance, written or verbal, and if verbal, how it was documented and witnessed). If your study included minors, state whether you obtained consent from parents or guardians. If the need for consent was waived by the ethics committee, please include this information.**

We have included information on participants’ written consent via an online survey and oversight via the University of Michigan IRB.

3. **Thank you for stating in your Funding Statement:**

**( This study was supported by grants from the National Institute of Mental Health (R01MH101459) to SS. JC was supported by T32HD007109 from the National Institute of Child Health and Development. The funders had no role in study design, data collection and analysis, decision to publish, or preparation of the manuscript.)**

**Please provide an amended statement that declares *all* the funding or sources of support (whether external or internal to your organization) received during this study, as detailed online in our guide for authors at** [**http://journals.plos.org/plosone/s/submit-now**](http://journals.plos.org/plosone/s/submit-now)**. Please also include the statement “There was no additional external funding received for this study.” in your updated Funding Statement.**

**Please include your amended Funding Statement within your cover letter. We will change the online submission form on your behalf.**

We have updated our Funding Statement and include it in the cover letter.

4. **In your Data Availability statement, you have not specified where the minimal data set underlying the results described in your manuscript can be found. PLOS defines a study's minimal data set as the underlying data used to reach the conclusions drawn in the manuscript and any additional data required to replicate the reported study findings in their entirety. All PLOS journals require that the minimal data set be made fully available. For more information about our data policy, please see** [**http://journals.plos.org/plosone/s/data-availability**](http://journals.plos.org/plosone/s/data-availability)**.**

**Upon re-submitting your revised manuscript, please upload your study’s minimal underlying data set as either Supporting Information files or to a stable, public repository and include the relevant URLs, DOIs, or accession numbers within your revised cover letter. For a list of acceptable repositories, please see** [**http://journals.plos.org/plosone/s/data-availability#loc-recommended-repositories**](http://journals.plos.org/plosone/s/data-availability#loc-recommended-repositories)**. Any potentially identifying patient information must be fully anonymized.**

**Important: If there are ethical or legal restrictions to sharing your data publicly, please explain these restrictions in detail. Please see our guidelines for more information on what we consider unacceptable restrictions to publicly sharing data:** [**http://journals.plos.org/plosone/s/data-availability#loc-unacceptable-data-access-restrictions**](http://journals.plos.org/plosone/s/data-availability#loc-unacceptable-data-access-restrictions)**. Note that it is not acceptable for the authors to be the sole named individuals responsible for ensuring data access.**

**We will update your Data Availability statement to reflect the information you provide in your cover letter.**

We have included the minimal underlying data set as a Supporting Information file in this revision.

5. **Thank you for stating the following in the Acknowledgments Section of your manuscript:**

**(This study was supported by grants from the National Institute of Mental Health (R01 MH101459) to S.S. and an investigator grant from Precision Health Initiative at University of Michigan, Ann Arbor to Z.W. and S.S.. J.C. was supported by T32HD007109. We thank the interns and residency programs who took part in this study.)**

**We note that you have provided funding information that is not currently declared in your Funding Statement. However, funding information should not appear in the Acknowledgments section or other areas of your manuscript. We will only publish funding information present in the Funding Statement section of the online submission form.**

**Please remove any funding-related text from the manuscript and let us know how you would like to update your Funding Statement. Currently, your Funding Statement reads as follows:**

**( This study was supported by grants from the National Institute of Mental Health (R01MH101459) to SS. JC was supported by T32HD007109 from the National Institute of Child Health and Development. The funders had no role in study design, data collection and analysis, decision to publish, or preparation of the manuscript.)**

**Please include your amended statements within your cover letter; we will change the online submission form on your behalf.**

Thank you for bringing this to our attention. We have removed the funding information from the Acknowledgements section and include the updated funding statement above.
