# Supplementary material for: A caveat to using wearable sensor data for COVID-19 detection: The role of behavioral change after receipt of test results
Source: PLoS One. 2022 Dec 30;17(12):e0277350. doi: 10.1371/journal.pone.0277350 (PMC9803125; doi:10.1371/journal.pone.0277350)
Supplement: Attachment — Submitted filename: ResponseToReviewers_PLOSONE_RR2.docx [file pone.0277350.s003.docx]

**Reviewer 2:**

1. **Consider this paper Clinical and Laboratory approach to Diagnose COVID-19 using Machine Learning, Interdisciplinary Sciences: Computational Life Sciences, Intelligent Computing on Time-Series Data Analysis and Prediction of COVID-19 Pandemics, Pattern Recognition Letters. Add future scope.**

We appreciate the reviewer’s suggestions of additional papers to review and appreciated their background on machine learning methods; these papers do focus on aspects of machine learning applications in detecting COVID-19 but in blood samples and social networking sites, respectively. Our analysis focuses on the changes in resting heart rate, physical activity, and sleep as measured by smartwatch/wearable technology during COVID-19 infection before and after receiving PCR test results. As our paper focuses primarily on behavioral change with test result date inclusion and less on developing a prediction algorithm or model, we restrained our literature review to those papers directly related to wearables metrics and change with COVID-19 infection. Additionally, we have incorporated text to better situate our paper in the existing literature and suggest future directions in Line 292 of the Discussion (see below).

**Reviewer 3:**

1. **The manuscript has largely improved compared to the previous version. The reviewers' suggestions were accepted and implemented in an appropriate way by the authors. The overall result was an increase in the quality of reporting. However, I think it is necessary to argue in more detail how the results of the article are consistent with the rest of the literature (line 313 of the revised manuscript), in order to discuss in depth the results of the original article in the light of the existing literature.**

Thank you for your comments on our revisions and direction to expand on our results in context. We have added a discussion of where our paper fits within a review of the wearables and infection detection literature and papers conducting similar analyses to ours.

Line 292-299: “To this end, changes in wearables data may not be fully able to serve as an early warning sign signaling individuals to seek COVID-19 testing; however, as the symptom onset versus positive antigen testing window changes with variants, this remains an important area for continued study. Additionally, as studies that incorporate other metrics such as self-reported symptoms(6,9) report additional discriminative capacity, objective but passively-collected measures of symptoms such as blood oxygen levels or body temperature may provide interesting new directions for detection algorithms.”
